# Screen Time Among Under-Five Children in India: A Systematic Review and Meta-Analysis

**DOI:** 10.7759/cureus.85458

**Published:** 2025-06-06

**Authors:** Ashish W Khobragade, M Swathi Shenoy

**Affiliations:** 1 Community and Family Medicine, All India Institute of Medical Sciences, Raipur, Raipur, IND

**Keywords:** children, meta-analysis, screen-based media, screen time, under-five

## Abstract

Digitalization, along with easy access and affordability to screen-based devices, has increased their use across all sectors of society. Due to the increased use of these devices among children, outdoor games have become less prevalent. The physical, mental, and social development of children may be adversely affected by excessive screen usage. The World Health Organization recommends limiting the screen time of under-five children.

The articles were searched for in PubMed, Scopus, and Google Scholar databases. The studies were screened and presented graphically using a Preferred Reporting Items for Systematic Reviews and Meta-Analyses (PRISMA) flowchart. Ten studies were used to determine the pooled screen time among under-five children in India. The study was registered in the International Prospective Register of Systematic Reviews (PROSPERO) with the registration ID CRD42023464810.

The pooled daily screen time among under-five children was 2.22 hours (95% CI: 1.80-2.63), with an I^2^ value of 100%. Due to the high heterogeneity, we employed a random-effects model to estimate the pooled screen time.

Under-five children in India are exposed to more screen time each day than is advised. The harmful consequences of too much screen time must be explained to parents.

## Introduction and background

Screen devices have become increasingly commonplace in recent years, drawing the interest of even the youngest fellow. Increased use of media devices affects a child's cognitive, social, and psychomotor development [1]. Guidelines restrict the maximum allowed sedentary screen time per day among children under five years of age. According to the World Health Organization, screen time is not recommended for children under one year of age, and not more than one hour is recommended for children between two and four years of age [2]. These guidelines also emphasize that "less is better" for optimal growth and development.

Determining the average time children under five spend in front of screens is essential to understanding how screen-based devices affect their development. Determining how much time young children spend in front of screens daily provides valuable insights into their routines and habits. It helps parents, educators, and legislators understand the advantages and disadvantages of screen time.

Many studies have been conducted in India on screen-based device use among under-five children, but the overall daily usage remains unknown. Assessing mean screen time will help develop and implement targeted preventive strategies.

The study aimed to find the pooled mean daily screen time among under-five children in India.

## Review

Materials and methods

We independently searched the articles in the PubMed, Scopus, and Google Scholar databases using the Patient/Population, Intervention, Comparison, and Outcome (PICO) criteria. The population was taken as under-five children in India. There was no intervention or comparator group. The outcome was the daily mean screen time. For the meta-analysis, we considered cross-sectional studies published in the English language. Studies conducted only in India were included. We restricted the search strategy period to the past five years.

PubMed Search Strategy

Articles were searched from PubMed using keywords and Boolean operators ‘AND’ and ‘OR’ (Screen time OR screen-based media) AND (child* OR infant* OR toddler* OR pre-schooler*) AND (India).

Search Strategy for Google Scholar and Scopus

Articles were searched from Google Scholar and Scopus using the following keywords: "Screen time", "screen exposure", "social media", "screen view time", "media devices", "sedentary screen time", "screen-based media", "mobile phones", "Video games", "Computers", "laptops", "electronic gadgets", "Desktops", "Television", "Under-five", "pre-schoolers", "toddlers", "Infants", "children", "young children", and "India".

**Figure 1:**
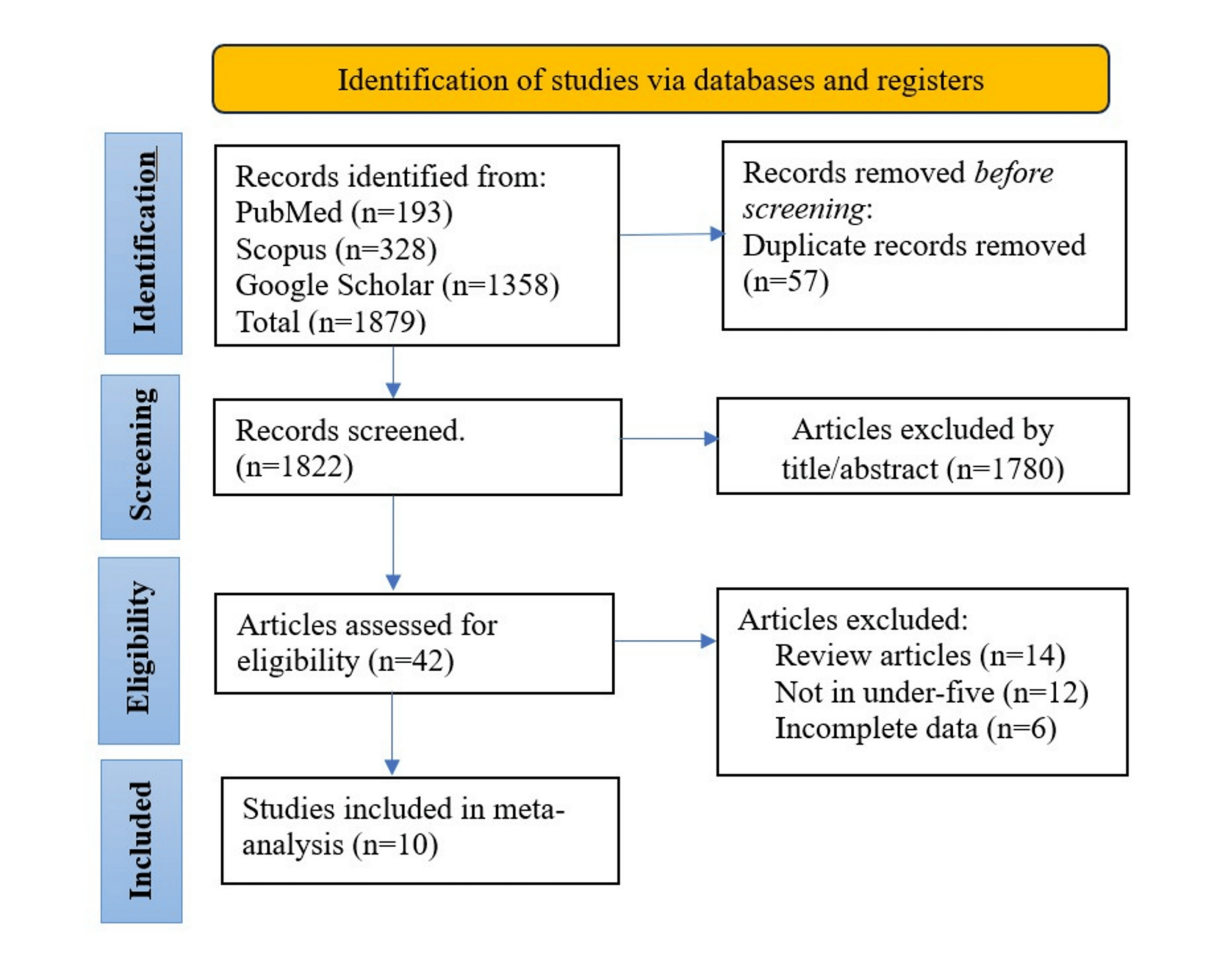
PRISMA flowchart PRISMA: Preferred Reporting Items for Systematic Reviews and Meta-Analyses

The articles meeting the inclusion criteria were selected. Duplicated articles were excluded. After the articles were selected, a full-text appraisal was done. Information on the region, year of publication, age of the children, total sample size, mean daily screen time, and standard deviation were extracted from each selected study. The selection of articles is illustrated using the Preferred Reporting Items for Systematic Reviews and Meta-Analyses (PRISMA) flowchart (Figure 1).

The Newcastle-Ottawa Scale (NOS), adapted for use in cross-sectional studies, was employed to evaluate study quality. It included sample selection, comparability, and outcome. Each component received a star rating, and the final score was calculated.

Statistical analysis was performed using the "meta" package of R software Version 4.3.2 (R Foundation for Statistical Computing, Vienna, Austria). The pooled analysis was presented in a forest plot. The pooled mean screen time was calculated using the inverse variance method and a random-effects model. Cochrane Q and Higgins and Thompson's I² statistics were calculated to determine the heterogeneity of the studies. A sensitivity analysis was performed by excluding each article once. Publication bias was graphically presented by a funnel plot. The asymmetry of the funnel plot was statistically tested using Egger's test and Begg and Mazumdar's test.

We conducted a subgroup analysis considering age, gender, year, and devices used. The study was registered in the International Prospective Register of Systematic Reviews (PROSPERO) with the registration ID CRD42023464810.

Results

We included 10 studies [3-12] in the meta-analysis, which comprise 2,857 children under the age of five years (Table 1).

**Table 1:** Descriptive characteristics of studies included for the meta-analysis of screen time among under-five children in India

Study	Year	Age group (years)	Region	Sample size	Newcastle-Ottawa Scale total score (stars)
Shirley and Kumar [3]	2019	2-5	Tamil Nadu	148	7
Krupa et al. [4]	2019	2-4	Tamil Nadu	46	6
Varadarajan et al. [5]	2021	0.5-5	Tamil Nadu	718	9
John et al. [6]	2021	2-5	Kerala	189	7
Gandhi and Oswal [7]	2021	2-5	Maharashtra	500	8
Kaur et al. [8]	2022	2-5	Chandigarh	400	8
Joseph et al. [9]	2022	0-3	Eastern India	57	6
Pasi et al. [10]	2022	1-5	Kerala	82	6
Agrawal et al. [11]	2022	2-5	Uttar Pradesh	299	7
Gayathri et al. [12]	2023	1-5	Tamil Nadu	418	8

**Figure 2:**
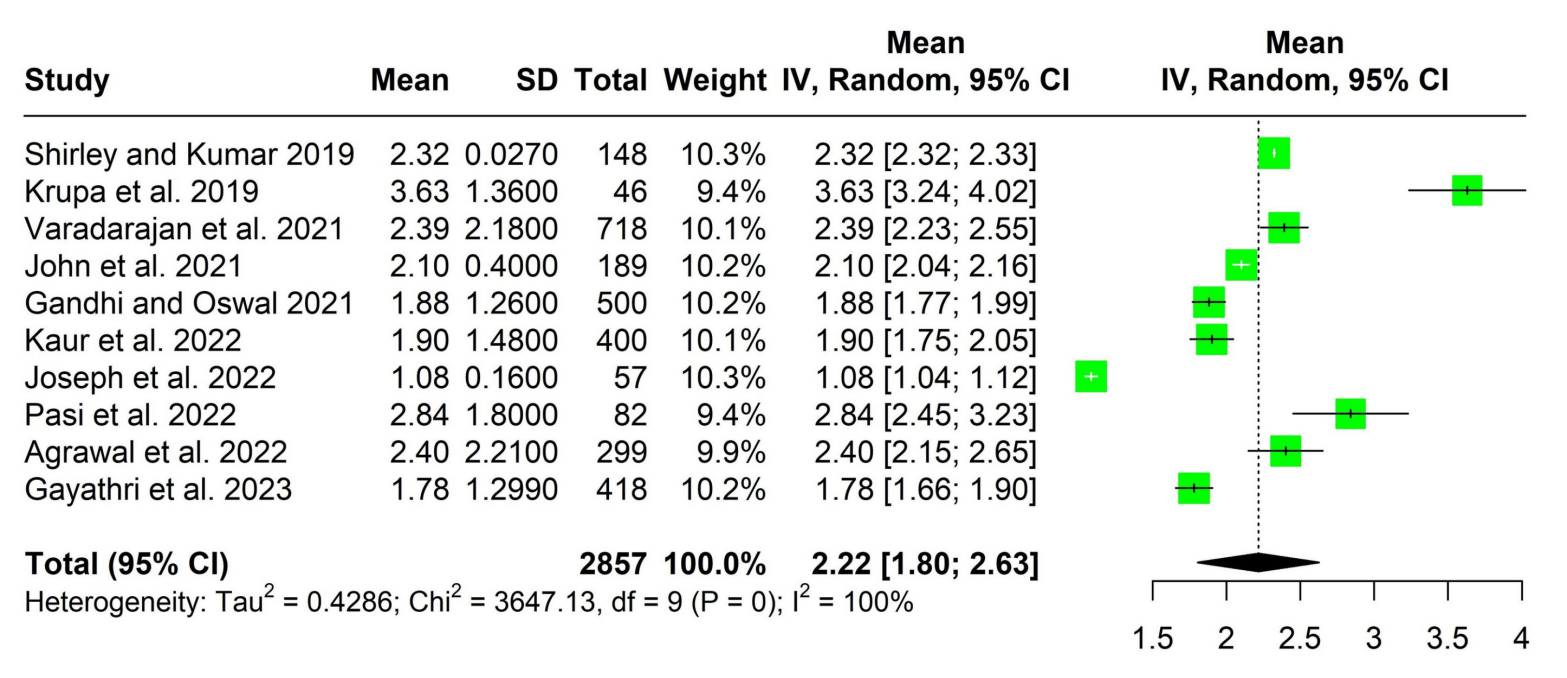
Forest plot showing pooled estimates of mean daily screen time among under-five children in India

The mean pooled daily screen time among under-five children in India was 2.22 hours (95% CI 1.80-2.63) with an I^2^ value of 100% (Figure 2).

We conducted a subgroup analysis to determine the mean daily screen time among children under the age of two years. We included two studies with a total sample size of 292. The pooled screen time among children under two years old was 1.23 hours (95% CI 1.08-1.39 hours), with an I^2^ value of 0%. The pooled estimates for this subgroup analysis were identical, obtained using both random- and common-effects models. We could not conduct other subgroup analyses due to the unavailability of the data.

We conducted a sensitivity analysis by excluding each study once; pooled estimates did not differ by more than 0.05 hours.

**Figure 3:**
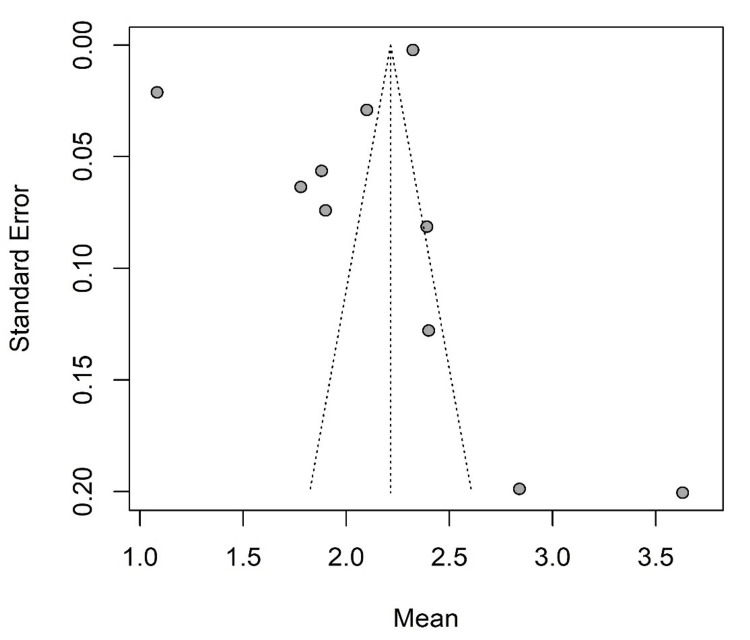
Funnel plot

Egger's and Begg and Mazumdar's tests were used to test publication bias. Egger's test gave a p-value of 0.261 (t=-1.21; df=8). Begg and Mazumdar's test gave a p-value of 0.089 (z=1.70), and after doing continuity correction, the p-value changed to 0.107 (z=1.61). These test results showed no evidence of publication bias. A funnel plot graphical presentation yielded similar findings (Figure 3).

Discussion

According to our study, under-five children spent an average of 2.22 hours a day (95% CI: 1.80-2.63 hours) in front of a screen, surpassing the recommended threshold. According to the Indian Academy of Pediatrics (IAP) guidelines, children between the ages of two and five years should ideally limit their screen time to less than one hour per day. Additionally, upon conducting subgroup analysis, we noted that the mean screen time for children under two years of age was 1.23 hours (95% CI: 1.08-1.39 hours). IAP guidelines stipulate that screen use should be completely avoided for this age range [13].

Alongside the time restrictions, the American Academy of Pediatrics recommends limiting overall screen time exposure and encouraging high-quality educational content when screen-based activities are permitted [14]. This approach aims to promote healthy development and well-being in young children while minimizing the potential negative impacts of excessive screen time across various media formats.

The early childhood landscape has undergone significant changes due to the widespread use of computers, tablets, cell phones, and televisions, prompting concerns about their long-term effects on children. According to research, early childhood screen use can have multifaceted implications of adverse impact on kids' physical, cognitive, and socioemotional development [15]. Increased screen usage has been linked to slowed language development, decreased cognitive function, and hampered social skill development [16,17]. In addition, an increased risk of obesity, disturbed sleep habits, and concentration issues have all been related to excessive screen time. To promote alternative activities that support holistic development and limit their children's screen time, parents and other caregivers play a crucial role. The studies we reviewed show a substantial correlation between screen time and parental screen time. To mitigate the negative impacts of excessive screen time, it is crucial to create tech-free zones inside the family, set clear and consistent screen time limits [18], and actively participate in offline play and interactions. Furthermore, promoting responsible digital citizenship from a young age can be achieved by setting a good example of screen habits and offering guidance on appropriate content [19,20].

Several stakeholders must collaborate to address the issues raised by excessive screen usage in children under five. Policymakers, educators, and medical experts must raise awareness about the possible dangers of excessive screen time and implement evidence-based techniques to mitigate its detrimental effects. Supporting kids' healthy development in the digital age requires encouraging outdoor play, fostering meaningful social connections, and promoting a balanced approach to technology use.

By prioritizing holistic well-being and mindful screen practices, we can create an environment that empowers children to thrive online and offline.

Limitations

Due to the unavailability of the data, we were unable to conduct a subgroup analysis of screen time according to different devices. Additionally, the studies included in the meta-analysis were from a limited number of states, which may introduce bias. Although the number of studies eligible for the meta-analysis was smaller, they provided strong evidence that daily screen time has increased among children under five.

## Conclusions

Screen time among under-five children in India exceeds the prescribed limit. Parents should limit their child's screen exposure while feeding or when crying. This helps to prevent the prolonged harmful effects on the child's development. Healthcare professionals should take the lead in educating people about the importance of using proper digital content and limiting children's exposure to screen time. Similar studies should be conducted in other countries to determine the mean daily screen time among under-five children to formulate country-specific guidelines.
